# Identification of miRNAs Responsive to *Botrytis cinerea* in Herbaceous Peony (*Paeonia lactiflora* Pall.) by High-Throughput Sequencing

**DOI:** 10.3390/genes6030918

**Published:** 2015-09-18

**Authors:** Daqiu Zhao, Saijie Gong, Zhaojun Hao, Jun Tao

**Affiliations:** Jiangsu Key Laboratory of Crop Genetics and Physiology, College of Horticulture and Plant Protection, Yangzhou University, Yangzhou 225009, China; E-Mails: dqzhao@yzu.edu.cn (D.Z.); gong.sai.jie@163.com (S.G.); haozhaojunko@126.com (Z.H.)

**Keywords:** herbaceous peony, *Botrytis cinerea*, miRNAs, target genes

## Abstract

Herbaceous peony (*Paeonia lactiflora* Pall.), one of the world’s most important ornamental plants, is highly susceptible to *Botrytis cinerea*, and improving resistance to this pathogenic fungus is a problem yet to be solved. MicroRNAs (miRNAs) play an essential role in resistance to *B. cinerea*, but until now, no studies have been reported concerning miRNAs induction in *P. lactiflora*. Here, we constructed and sequenced two small RNA (sRNA) libraries from two *B. cinerea*-infected *P. lactiflora* cultivars (“Zifengyu” and “Dafugui”) with significantly different levels of resistance to *B. cinerea*, using the Illumina HiSeq 2000 platform. From the raw reads generated, 4,592,881 and 5,809,796 sRNAs were obtained, and 280 and 306 miRNAs were identified from “Zifengyu” and “Dafugui”, respectively. A total of 237 conserved and 7 novel sequences of miRNAs were differentially expressed between the two cultivars, and we predicted and annotated their potential target genes. Subsequently, 7 differentially expressed candidate miRNAs were screened according to their target genes annotated in KEGG pathways, and the expression patterns of miRNAs and corresponding target genes were elucidated. We found that miR5254, miR165a-3p, miR3897-3p and miR6450a might be involved in the *P. lactiflora* response to *B. cinerea* infection. These results provide insight into the molecular mechanisms responsible for resistance to *B. cinerea* in *P. lactiflora*.

## 1. Introduction

Herbaceous peony (*Paeonia lactiflora* Pall.) is a perennial root flower belonging to the Paeoniaceae family. It is popular worldwide due to its high ornamental values in locations including New Zealand, Australia, Europe, Asia, and North America [1]. In China, *P. lactiflora* has been widely applied in urban and rural landscaping due to its high levels of resistance to infection, tolerance to environmental factors, and easy maintenance, making it ideal for a variety of specialized gardens, flower beds, and perennial borders [2]. However in practice, *P. lactiflora* cultivated in a landscape greenbelt in the middle and lower reaches of the Chinese Yangtze River region was highly susceptible to *Botrytis cinerea* after flowering. This infection mainly damaged the *P. lactiflora* leaf and resulted in rot and led to the death of the entire plant, which was consistent with the previous findings in Nanjing, Shanghai and Hangzhou [3]. Lan [3] reported that the optimum temperature for *B. cinerea* growth and sporulation was 20 °C–24 °C and the optimum pH was 4–5, and it could be controlled by many chemical treatments. However, chemical spraying is expensive, requires manual labor, pollutes the environment, and leaves white stains on the leaves, ultimately reducing the ornamental value of *P. lactiflora* while not necessarily preventing *B. cinerea* infection. Conversely, breeding resistant cultivars is the best method to solve this problem, and the rapid development of genetic engineering technology in recent years has laid the foundation for effective development of this field. Recent studies have provided compelling evidence demonstrating that microRNAs (miRNAs) are essential endogenous regulatory factors and play an important role in plant response to biotic stress [4,5]. Analysis of miRNAs thus provides a novel method to study the molecular mechanism of resistance in *P. lactiflora B. cinerea*.

miRNAs are approximately 21-nucleotides (nt) in length and are non-coding small RNAs (sRNAs) found in animals and plants, typically encoded by endogenous genes. miRNAs could play an important regulatory role at the post-transcriptional level by targeting mRNA degradation and leading to translation repression [6]. Since they were first found in *Caenorhabditis elegans* [7], a large number of miRNAs have been continually identified in higher plants, including *Arabidopsis thaliana* [5], *Zea mays* [8], *Triticum aestivum* [9] and *Oryza sativa* [10]. The latest version of the miRNA database (miRBase 21.0, http://www.mirbase.org/) contains 28,645 entries of hairpin precursor miRNAs (pre-miRNAs), expressing 35,828 mature miRNA products over a range of 223 species [11]. Moreover, numerous studies have revealed that miRNAs are involved in diverse biological and metabolic processes, such as the regulation of plant organ development [12,13], signal conduction of plant hormones [14,15], abiotic stress response [16,17], and pathogen defense [18]. To the best of our knowledge concerning *B. cinerea*, only Jin *et al.* [19,20] have reported that tomato miRNAs had a functional role in resistance to *B. cinerea*. However, there no *P. lactiflora* miRNAs have yet been deposited in miRBase and no related reports on this topic have been completed. In order to investigate the roles of *P. lactiflora* miRNAs in response to *B. cinerea* stress, two cultivars, “Zifengyu” and “Dafugui,” with significantly different levels of resistance to this pathogen were processed for sRNA and transcriptome sequencing. In this study, miRNAs and their target genes responsive to *B. cinerea* were identified in *P. lactiflora*, and these results provide a foundation for understanding the functions and regulatory mechanisms of miRNAs in *P. lactiflora* resistance to *B. cinerea*.

## 2. Experimental Section

### 2.1. Plant Materials

**Figure 1:**
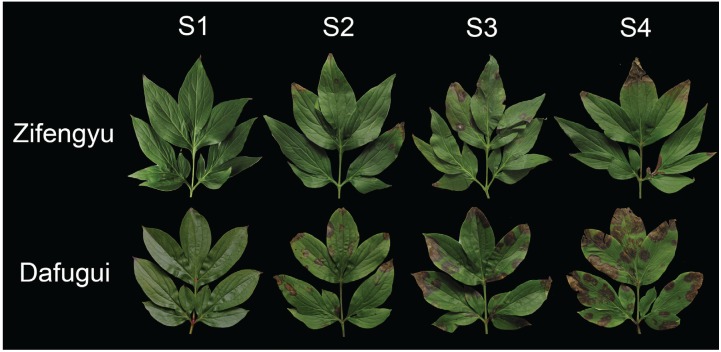
The infection status of the cultivars “Zifengyu” and “Dafugui” under field conditions by *B. cinerea*. S1, late May; S2, mid June; S3, early July; and S4, late July.

*P. lactiflora* was grown in the germplasm repository of Horticulture and Plant Protection College, Yangzhou University, Jiangsu Province, China (32°30' N, 119°25' E). Under field conditions, two cultivars, “Zifengyu” and “Dafugui”, with significantly different levels of resistance to *B. cinerea* (Figure 1, in the later stage infected by *B. cinerea*, the resistant cultivar “Zifengyu” grew well with few disease spots, while the susceptible cultivar “Dafugui” became weak and almost entirely withered) were used as the experimental materials for sRNA and transcriptome sequencing. After flowering, *P. lactiflora* was naturally infected by *B. cinerea*, and the infected and fully expanded leaves from the fourth apical node in four developmental stages (S1, late May; S2, mid June; S3, early July; and S4, late July) were taken from May to July 2013. The sample population of each cultivar were sixty plants (four stages, fifteen plants each stage). The leaves collected from four stages each cultivar were then equally mixed to prepare for two independent sRNA libraries (*i.e.*, “Zifengyu” and “Dafugui”). All samples were immediately frozen in liquid nitrogen and stored at −80 °C until further analysis.

### 2.2. Small RNA Library Construction and Sequencing

Total RNA was extracted according to a modified CTAB extraction protocol [21]. Prior to sRNA library construction, RNA samples were examined by a spectrophotometer (Eppendorf, Hamburg, Germany) and 1% agarose gel electrophoresis. Moreover, RNA fragments of 18–30 nt long were separated from total RNA using polyacrylamide gel electrophoresis. The Solexa adaptors were added to the fragments at both 5'- and 3'-ends, and they were converted to cDNA according to a reverse transcription PCR kit (Invitrogen, Carlsbad, CA, USA). The purified fragments were sent for sequencing at the Beijing Genomic Institute (Shenzhen, China) using the Illumina HiSeq 2000 platform (Illumina Inc., San Diego, CA, USA). The data from “Zifengyu” and “Dafugui” were submitted to the National Center for Biotechnology Information (NCBI) under accession numbers SRS926009 and SRS926051, respectively.

### 2.3. miRNA Identification and Corresponding Target Gene Prediction

After getting rid of low-quality sequences (reads with 5' primer contaminants, reads without 3' primer, reads without the insert tag, reads with poly A and reads shorter than 18 nt), unique reads were screened against GenBank as well as Rfam (http://rfam.sanger.ac.uk) to remove ribosomal RNA (rRNA), transfer RNA (tRNA), small nuclear RNA (snRNA), and small nucleolar RNA (snoRNA). Moreover, the common and specific sequences between two libraries were identified through the comparison of corresponding genes annotated by transcripts. After these, the merged unique reads were also screened against the miRBase 21.0 using a nucleotide-nucleotide Basic Local Alignment Search Tool (BLASTn) to identify the conserved miRNAs [11]. Meanwhile, in order to identify the novel miRNAs, all candidate precursors with hairpin-like structures were obtained using the Mireap program (http://sourceforge.net/projects/mireap). Additionally, the unigene sequences of “Zifengyu” and “Dafugui” transcriptomes submitted to the NCBI with accession numbers SRS774325 and SRS774327 were used to predict the target genes of miRNAs by the psRNA Target program (http://plantgrn.noble.org/psRNATarget) with the default parameters. The specific methods were from Allen *et al.* [22] and Schwab *et al.* [23].

### 2.4. Differentially Expressed miRNAs and Their Target Gene Annotation

miRNAs expression levels were calculated according the value of reads per million reads (RPM). Differentially expressed miRNAs were defined based on strict criteria (*p* value ≤ 0.05 and differential expression fold > 2). Functional annotation of target genes was performed using various bioinformatics procedures, including GO and KEGG. The specific methods were referred to in Gong *et al*. [24].

### 2.5. Digital Gene Expression Analysis

Firstly, eight cDNA libraries from “Zifengyu” and “Dafugui” in four developmental stages were constructed. After quantification and qualification of the sample library using the Agilent 2100 Bioanalyzer (Agilent Technologies, Palo Alto, CA, USA) and ABI StepOnePlus Real-Time PCR System (Applied Biosystems, Foster, California, CA, USA), the samples were also sequenced using the Illumina HiSeq 2000 platform (Illumina, San Diego, CA, USA). The gene expression level was compared with S1, respectively (S1/S1, S2/S1, S3/S1, S4/S1), and calculated using the reads per kb per million reads (RPKM) method [25] based on the numbers of reads uniquely mapped to the specific gene and the total number of uniquely mapped reads in the sample.

### 2.6. Expression Analysis of Target Genes by qRT-PCR

Expression levels of selected target genes were analyzed by quantitative real-time PCR (qRT-PCR) with three biological replications each sample via a CFX96 Real-Time System (Bio-Rad, Hercules, CA, USA). The specific methods were referred to in Zhao *et al*. [26]. The cDNA was synthesized from 1 g RNA using PrimeScript RT reagent Kit With gDNA Eraser (TaKaRa, Kyoto, Japan). *P. lactiflora* Actin (JN105299) had been used as an internal control in this study [27]. Gene-specific primers were designed using PRIMER5.0 software and listed in Table S1. Two µL of the cDNAs of each sample were used for ordinary PCR to test the amplification specificity of the corresponding primer pairs. qRT-PCR was performed using the SYBR Premix Ex Taq (Perfect Real Time) (TaKaRa). The amplification system consisted of an initial denaturation of 95 °C/30 s, followed by 40 cycles of 95 °C/5 s, 51 °C/30 s, 72 °C/30 s. Gene relative expression levels were calculated by the 2^−∆∆Ct^ comparative threshold cycle (Ct) method [28]. The Ct values of the triplicate reactions were gathered using the Bio-Rad CFX Manager V1.6.541.1028 software (Bio-Rad, Hercules, CA, USA, 2008).

## 3. Results

### 3.1. Sequence Analysis of sRNAs

**Figure 2:**
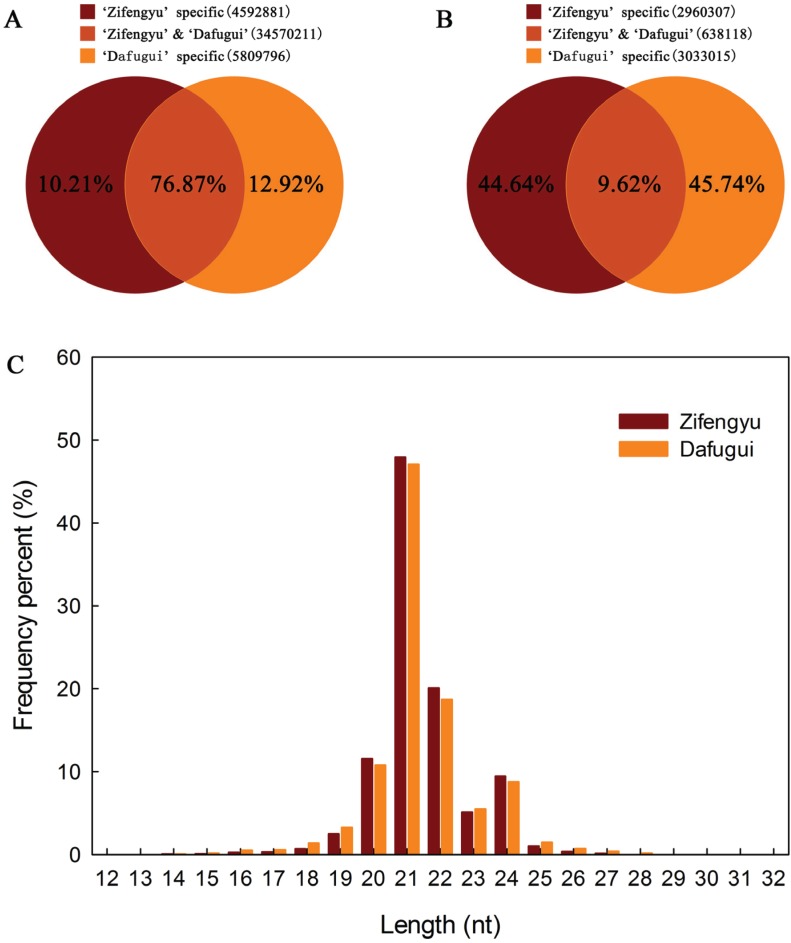
Venn diagrams for analysis of total (**A**) and unique (**B**) sRNAs from “Zifengyu” and “Dafugui” libraries, and the length distribution of sRNAs (**C**).

To identify miRNAs responsive to *B. cinerea* in *P. lactiflora*, two independent sRNA libraries were sequenced from different cultivars collected at four developmental stages and equally mixed, both subject to *B. cinerea* infection, but one of which (“Zifengyu”) is resistant to the pathogen. A total of 24,008,974 and 22,108,093 reads were generated from “Zifengyu” and “Dafugui”, respectively. The low quality sequences were removed including 5' contaminants, those missing the 3' primer or insert tag, sequences with a poly A tail, and finally those shorter than 18 nt. The final data sets consisted of 23,520,582 and 21,452,306 clean reads in “Zifengyu” and “Dafugui”, respectively, which was in both cases more than 97.97% of the total original reads. Subsequent analysis revealed the number of total sRNAs (*i.e.*, sum of sRNAs) was 4,592,881 in “Zifengyu” and 5,809,796 in “Dafugui” (Figure 2A), while the number of unique sRNAs (*i.e.*, variety of sRNAs) was 2,960,307 and 3,033,015, respectively (Figure 2B). Moreover, the length distribution of sRNA was similar between the two libraries, where the sRNAs ranging from 21 to 24 nt was the most frequent length (more than 80%) identified, c (more than 47%), followed by 22 nt, 20 nt, and 24 nt (Figure 2C). These sRNAs were classified into different categories after screening against GenBank and Rfam using BLAST searches, and the results from these analyses are presented in Table 1.

**Table 1:** Distribution of sRNAs among different categories in “Zifengyu” and “Dafugui”.

Type	Unique sRNAs		Total sRNAs	
	“Zifengyu”	“Dafugui”	“Zifengyu”	“Dafugui”
rRNA	60653	77278	1047570	1338595
tRNA	16257	19165	1180850	1206549
snRNA	4057	4310	16627	15686
snoRNA	1031	1033	3289	3180
Unannotated	3496291	3540283	19428764	17158589
miRNA	20136	29064	1843482	1729707
Total	3598425	3671133	23520582	21452306

### 3.2. Identification of Conserved miRNAs

To identify conserved miRNAs, all mappable sRNA sequences were compared with the known plant miRNAs in miRBase. A total of 271 and 298 conserved miRNA sequences were identified in “Zifengyu” and “Dafugu”, respectively (Tables S2 and S3). The read counts among different miRNA families were markedly different. A few conserved miRNA families such as miR5078, miR166a, miR167a, miR157a, miR6113, miR718, miR1882e-3p, miR3353 and miR8019-5p were enriched in both of the libraries. The secondary structures of some conserved pre-miRNAs in “Zifengy” and “Dafugu” are listed in Figures S1 and S2, respectively. Moreover, putative target genes of conserved miRNAs were predicted, with 406 and 458 potential target genes from 133 (out of 271) and 136 (out of 298) conserved miRNAs identified in “Zifengy” and “Dafugu”, respectively (Tables S4 and S5).

### 3.3. Identification of Novel miRNAs

After searching for potential pre-miRNAs and predicting their hairpin-like structures, 9 and 8 unique sequences were identified as novel miRNAs in “Zifengyu” and “Dafugui”, respectively (Table 2 and Table 3), only 2 of which (pla-MIR11601 and pla-MIR11605) were shared by both cultivars. The novel miRNA sequences were 20 to 23 nt in length, and among these 21 nt reads were the most abundant. The pre-miRNAs ranged from 76 to 262 nt in “Zifengyu” and 76 to 274 nt in “Dafugui” (Table 2 and Table 3) in length. We also obtained the secondary structures of select novel pre-miRNAs (Figure S3). The average minimum free energy values were −41.31 kcal/mol in “Zifengyu” and −42.35 kcal/mol in “Dafugui”. Furthermore, we predicted the putative target genes of novel miRNAs were predicted, with 12 and 40 potential target genes from 8 (out of 9) and 7 (out of 8) novel miRNAs identified in “Zifengyu” and “Dafugui”, respectively (Tables S6 and S7).

**Table 2:** Novel miRNA candidates in “Zifengyu”.

Name in miRBase	Sequence	Length (nt)	Start/End Precursor	Length of Precursor (nt)	Arm	MFE (kcal/mol)	Count
pla-MIR11601	TGCTCTAAAAGATCGTAGTTC	21	1–262	262	5'	−34.80	12
pla-MIR11605	TTGAGGCGGCATATTCTCAAT	21	73–178	106	3'	−29.82	18
pla-MIR11606	TGGTGGACTCCAATTCGCATA	21	1563–1694	132	3'	−49.60	1113
pla-MIR11607	GATCACTCGGTTGTCTGACACAC	23	169–297	129	5'	−27.50	1258
pla-MIR11608	TCGCTTAGGGGTTGTTGAAGCGC	23	119–222	104	5'	−38.60	29
pla-MIR11609	TAGCTTGGTGTGAGGTCAACTT	22	132–309	178	5'	−43.50	11
pla-MIR11610	ACAGATATGGTAGGGGGCACA	21	10,914–10,989	76	3'	−36.70	222
pla-MIR11611	TAGGCAACCGTGGTAAAATGTC	22	945–1045	101	3'	−48.10	6
pla-MIR11612	AAGACGGTCCAAAACGCCCAC	21	151–283	133	3'	−63.20	490

**Table 3:** Novel miRNA candidates in “Dafugui”.

Name in miRBase	Sequence	Length (nt)	Start/End Precursor	Length of Precursor (nt)	Arm	MFE (kcal/mol)	Count
pla-MIR11598	TCGTTCAAAGTAGGTTGTCAA	21	113–349	237	5'	−45.60	16
pla-MIR11599	TTCAACCGTGGTAGATGTTAA	21	95–306	212	5'	−92.70	13
pla-MIR11600	AACGTTCCTCGATTTCGCGAT	21	471–546	76	3'	−24.90	6
pla-MIR11601	TGCTCTAAAAGATCGTAGTTC	21	1–262	262	5'	−34.80	15
pla-MIR11602	TCTAACGGAACGCTATTGGATC	22	6884–6998	115	3'	−22.10	8
pla-MIR11603	TTATAATTAGGTTGAGCGGAC	21	40–313	274	5'	−62.00	2979
pla-MIR11604	TCCAGAGGGAGAACGTGGCGA	21	328–406	79	3'	−26.90	9
pla-MIR11605	TTGAGGCGGCATATTCTCAAT	21	73–178	106	3'	−29.82	41

### 3.4. Comparative Analysis of miRNAs and Corresponding Target Genes between Two Libraries

**Figure 3:**
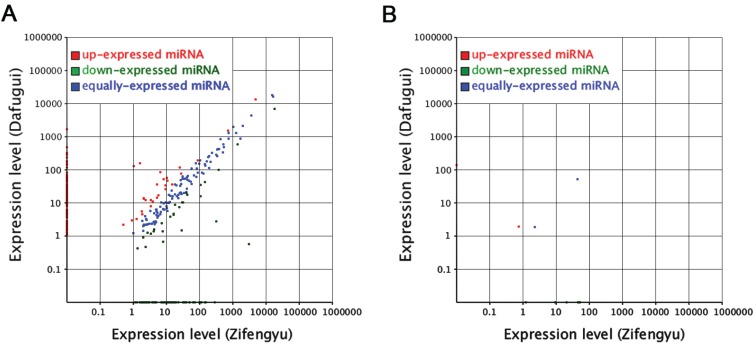
Differentially expressed conserved (**A**) and novel (**B**) miRNAs from “Zifengyu” and “Dafugui” libraries. Red scatters indicate up-regulated miRNAs, green scatters indicate down-regulated miRNAs, and blue scatters indicate no difference miRNAs in expression between “Zifengyu” and “Dafugui”.

To identify key miRNAs between the two *P. lactiflora* libraries, their conserved miRNAs were comparatively analyzed based on “Zifengyu” as the control group. A total of 237 differentially expressed miRNAs were obtained (Figure 3A and Table S8), and 136 miRNAs were up-regulated, whereas 101 miRNAs were down-regulated. We found that 436 potential target genes were identified from 115 (out of 237) differentially expressed miRNAs. In order to further evaluate the potential functions of these target genes, a Kyoto Encyclopedia of Genes and Genomes (KEGG) functional classification was performed. In the KEGG functional classification analysis, only 130 target genes could be assigned to 12 KEGG pathways, among which metabolic pathways demonstrated the largest number of target genes, followed by biosynthesis of secondary metabolites, endocytosis and ether lipid metabolism. In addition, some pathways closely related to plant resistance were found including plant hormone signal transduction, phenylpropanoid biosynthesis, and plant-pathogen interaction (Table S9).

Similarly, the novel miRNAs were also comparatively analyzed, and 9 differentially expressed miRNAs were identified, including 2 up-regulated and 5 down-regulated miRNAs (Figure 3B and Table S10). Among these sequences, 8 potential target genes were identified from 6 (out of 7) differentially expressed miRNAs. After KEGG functional classification, only 2 target genes could be assigned to 1 KEGG pathways (Table S11).

### 3.5. Analysis of the Candidate *B. cinerea*-Responsive miRNAs

In order to identify the miRNAs responsive to *B. cinerea* stress, the analyses of differentially expressed miRNAs and their target genes in two libraries were performed. According to the target genes annotated in KEGG pathways, 9 genes were screened that were linked to phenylpropanoid biosynthesis (beta-glucosidase, *BGLU*), diterpenoid biosynthesis (gibberellin 2-oxidase, *GA2OX*), plant hormone signal transduction (ethylene receptor, *ETR*), plant-pathogen interaction (nonhost1, *NHO1*; peroxin-10, *PEX10*; serine/threonine-protein kinase PBS1, *PBS1*; WRKY transcription factor 29, *WRKY29*; calcium-dependent protein kinase, *CDPK*). The corresponding miRNAs were miR5254, miR846, miR6450a, miR165a-3p, miR6432, miR6450a, miR5558-3p and miR3897-3p, respectively (Table 4). When “Zifengyu” was regarded as the control group, the expression levels of the majority of the candidate miRNAs in “Dafugui” were up-regulated, except for miR6450a and miR5558-3p. Furthermore, the expression patterns of 9 target genes were analyzed in four developmental stages using digital gene expression. The results showed that these genes were essentially up-regulated after infection by *B. cinerea*. Additionally, *BGLU*, *NHO1*, *CDPK* and *WRKY29* were highly expressed in “Zifengyu”, whereas the opposite trend was observed in *ETR*, which harbored expression levels negatively correlated with corresponding miRNAs (miR5254, miR165a-3p, miR3897-3p and miR6450a).

**Table 4:** Candidate *B. cinerea*-responsive miRNAs and their target genes.

miR-Name	Fold-Change (log2 Dafugui/Zifengyu)	Target Gene	Annotation	Expression Pattern of Target Gene (log2 Sn/S1)
miR5254	9.02815472	CL2508.Contig1_All	beta-glucosidase	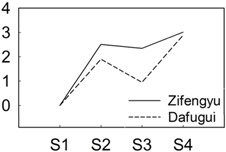
miR846	12.12015362	Unigene17406_All	gibberellin 2-oxidase	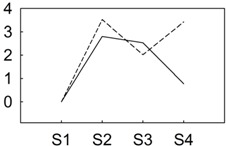
miR165a-3p	1.39849113	CL10731.Contig2_All	nonhost1	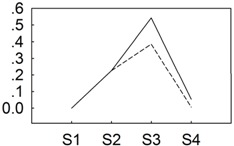
miR6432	8.82072147	CL7438.Contig1_All	peroxin-10	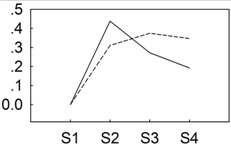
miR6450a	−11.19652845	Unigene9181_All	ethylene receptor	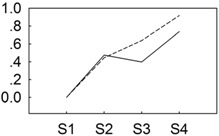
		CL6799.Contig1_All	serine/threonine-protein kinase PBS1	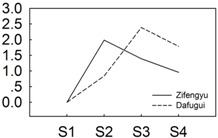
miR5558-3p	−2.35248612	CL8576.Contig1_All	serine/threonine-protein kinase PBS1	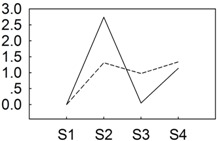
miR3897-3p	7.50620839	Unigene17579_All	calcium-dependent protein kinase	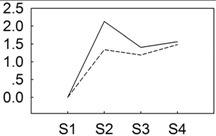
		Unigene3137_All	WRKY transcription factor 29	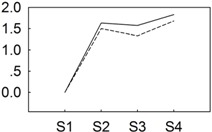

### 3.6. qRT-PCR Analysis of Target Genes

**Figure 4:**
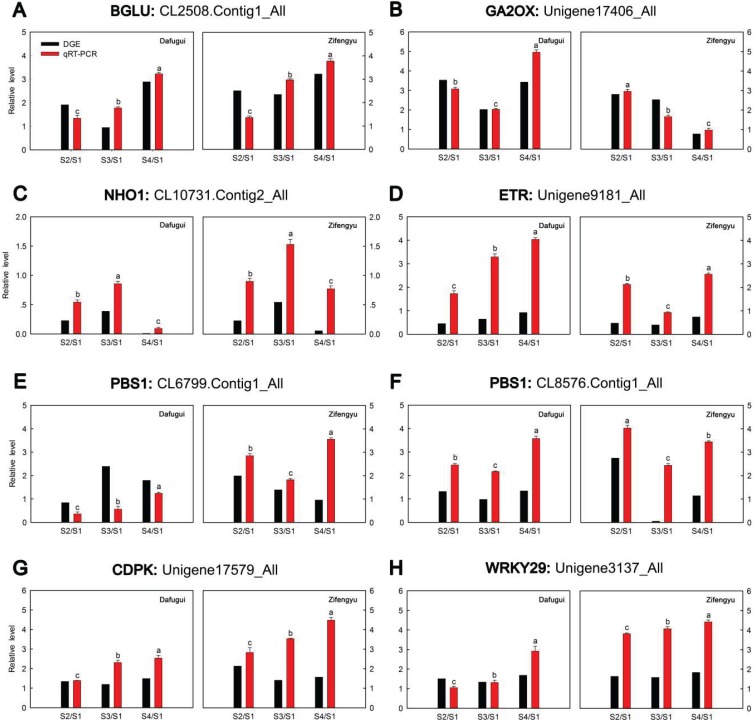
qRT-PCR validations of expression levels of target genes from digital gene expression analysis. Expression levels by qRT-PCR of selected target genes of *P. lactiflora* cultivars “Dafugui” and “Zifengyu” were validated from the levels of digital gene expression data. The corresponding genes are specified above each map. The Y axis represents the normalized log2 value of gene expression levels. The X axis represents the comparisons of different stages. “S2/S1” indicates a comparison of gene expression levels between S1 and S2. “S3/S1” and “S4/S1” indicate analogous comparisons. S1: late May; S2: mid June; S3: early July; S4: late July.

To verify the results of the digital gene expression analysis, expression levels of eight target genes including *BGLU* (CL2508.Contig1_All), *GA2OX* (Unigene17406_All), *NHO1* (CL10731.Contig2_All), *ETR* (Unigene9181_All), *PBS1* (CL6799.Contig1_All), *PBS1* (CL8576.Contig1_All), *CDPK* (Unigene17579_All), and *WRKY29* (Unigene3137_All) were evaluated by qRT-PCR (Figure 4). Overall, the relative expression of the examined target genes in “Zifengyu” and “Dafugui” at four different stages showed similar expression patterns according to the digital gene expression profiles, which indicated a correspondence of the results from qRT-PCR with the digital gene expression sequencing.

## 4. Discussion

It has been recently found that miRNAs play important roles in physiological processes as a non-coding gene expression and regulating factor [29]. With the dramatic changes in the environment, more attention has been paid to the protective role of miRNAs in plants. Response to adversity stress in plants includes the differential expression of miRNAs, causing the accumulation of select substances and altering metabolic pathways [4,5,30]. Plant adversity stress is divided into abiotic and biotic categories, with more in-depth studies focused on plant miRNAs in response to abiotic stress, which includes stresses related to changes in nutrition [31], water [32], temperature [33], and heavy metals [34]. Meanwhile, miRNAs also play an important role in response to biotic stress; in particular, when plants are infected by pathogens, the expression of a variety of miRNAs and related target genes will change [35,36]. In the present study, high-throughput sequencing technology was used to identify miRNAs responsive to *B. cinerea* infection in *P. lactiflora* for the first time. Two cultivars, “Zifengyu” and “Dafugui”, with significantly different levels of resistance to *B. cinerea* were selected as the materials. When they were subjected to *B. cinerea* infection, “Zifengyu” was resistant to the pathogen. The two plant cultivars with different genotypes were used as experimental materials and compared in terms of many stress responses, such as temperature stress response [32] and *Verticillium dahliae* and *Sporisorium reilianum* infection [37,38]. In *B. cinerea* infection, our group compared the digital gene expression (DGE) of “Zifengyu” and “Dafugui” subjected to *B. cinerea* infection, and a great deal of disease resistance-relevant genes were successfully screened [24]. Thus, these two cultivars might be good materials to study the functions and molecular mechanisms of miRNAs in *P. lactiflora* resistance to *B. cinerea.* Through comparing two independent sRNA libraries, 23,520,582 and 21,452,306 sRNAs in “Zifengyu” and “Dafugui” were obtained, respectively. The length distribution of sRNAs in the libraries was very similar with the majority ranging from 20 to 24 nt. Among these sequences, the 21 nt sRNAs were the most abundant, followed by 22 nt, which is consistent with reports for Chinese yew [39] and Norway spruce [40]. However, in reports for other plants, such as celery [33], trifoliate oranges [41], and olives [42], the 24 nt sRNAs were the most abundant. These differences are mainly because the length of sRNAs is dependent on specific enzymes. For example, the sRNAs are 21 nt in length when processed by DCL1, while they are 24 nt when processed by DCL2 [43]. These sRNAs were classified into different categories, with the rRNA and miRNA groups enriched, which is consistent with what has been found in winter wheat [44], suggesting that sRNAs have been conserved in plant evolution. In the comparative analysis of miRNAs between “Zifengyu” and “Dafugui”, 237 conserved and 7 novel differentially expressed miRNAs were obtained. We examined the KEGG pathways with which they were associated in order to distinguish those likely to be involved in resistance to *B. cinerea* from those related to other phenotypic differences between the two cultivars.

The molecular mechanistic changes in plants infected by *B. cinerea* are complex; however, previous studies mainly focused on the transcriptional level of this response. De Cremer *et al.* [45] analyzed the transcriptome of lettuce infected by *B. cinerea*, and identified a complex network of genes, including the induction of those related to the phenylpropanoid pathway, terpenoid biosynthesis, and photosynthesis. At the same time, through sequencing the tomato transcriptome, Smith *et al.* [46] found that almost immediately after *B. cinerea* infection, a variety of processes were suppressed including photosynthesis as well as pathways involved in growth, energy generation, and response to stimuli. Simultaneously, some processes were also induced such as various defense-related genes, including pathogenesis-related protein 1 (PR1), a beta-1, 3-glucanase (glucanase), and a subtilisin-like protease. At the post-translational level, the relevant reports concerning *B. cinerea* infection have been limited to the tomato. Firstly, Jin *et al.* [20] identified three *B. cinerea* stress-responsive miRNAs using microarray analysis, which regulated metabolic, morphological, and physiological adaptations of tomato seedlings at the post-transcriptional level. Subsequently, Jin and Wu [19] investigated the miRNA expression patterns in tomato in response to *B. cinerea* stress using high-throughput sequencing, and found that 57 conserved miRNAs and one novel miRNA were differentially expressed in *B. cinerea*-infected leaves, and additionally that miR319, miR394 and miRn1 might be involved in the tomato leaf response to *B. cinerea* infection. In the present study, 7 candidate differentially expressed miRNAs related to adversity stress were screened, and the 9 corresponding target genes were annotated in phenylpropanoid biosynthesis, diterpenoid biosynthesis, plant hormone signal transduction, and plant-pathogen interaction using KEGG. Among the candidate miRNAs, miR5254, miR846, miR165a-3p, miR6432 and miR3897-3p revealed higher expression levels in “Dafugui” than “Zifengyu”, while the conserved miRNAs, including miR6450a and miR5558-3p, had the opposite expression pattern. For the target genes analyzed, the expression levels of *BGLU*, *NHO1*, *CDPK*, and *WRKY29* in “Zifengyu” were always higher than those in “Dafugui”, whereas that of *ETR* was always lower. The expression levels of *PEX10* and *PBS1* were higher in “Zifengyu” than “Dafugui” at an early stage of infection (S2), but lower later during the infection (S3 and S4), while that of *GA2OX* was lower in “Zifengyu” than “Dafugui” at S2 and S4, but higher at S3. In previous studies, *BGLU* was associated with many biological processes in plants that could resist abiotic and biotic stresses by activating plant hormones and resistant pathways [47]; for example, overexpression of an *Arabidopsis* *BGLU* gene enhanced drought resistance in creeping bentgrass [48]. Lu *et al.* [49] found *NHO1* was required for general resistance against *Pseudomonas* bacteria in *Arabidopsis*. *CDPK* has multiple functions in plant disease resistance, such as enhancing the production of active oxygen species (AOS) by stimulating NADPH oxidase activity [50]. Additionally, as a member of the complex family of WRKY transcription factors, *WRKY29* has been proved to be a positive regulator of disease resistance [51], while *ETR* negatively regulated ethylene responses [52]. The expression levels of these 5 target genes in the present study were consistent with previous studies, which indicated that they might be involved in the *P.* *lactiflora* response to *B. cinerea* infection at the transcriptional level. Moreover, these 5 target genes were negatively correlated with their corresponding miRNAs (miR5254, miR165a-3p, miR3897-3p and miR6450a), which also suggested that they might be involved in the *P.* *lactiflora* response to *B. cinerea* infection at the post-transcriptional level. However, to the best of our knowledge concerning miRNAs, only miR165a was previously reported in response to abiotic stress [53], meaning the roles of miR5254, miR3897-3p and miR6450a in response to *B. cinerea* infection require further study. These results would further the understanding of miRNA regulation in response to *B. cinerea* stress in *P.* *lactiflora*.

## 5. Conclusions

In this study, high-throughput sequencing technology was used to characterize the miRNAs in the *B. cinerea*-infected two *P.* *lactiflora* cultivars “Zifengyu” and “Dafugui” with significantly different resistance to *B. cinerea*. After stringent quality checking and data cleaning, a total of 23,520,582 and 21,452,306 clean reads were obtained. Differential expression analysis revealed 237 conserved and 7 novel miRNAs between “Zifengyu” and “Dafugui” might be associated with *B. cinerea* stress resistance. Among screening, miR5254, miR165a-3p, miR3897-3p and miR6450a might be involved in the *P.* *lactiflora* response to *B. cinerea* infection. Our work was useful for breeding new cultivars of *B. cinerea* stress resistance.

## Supplementary Files

Supplementary File 1
