# Supplementary material for: Identification of miRNAs Responsive to Botrytis cinerea in Herbaceous Peony (Paeonia lactiflora Pall.) by High-Throughput Sequencing
Source: Genes (Basel). 2015 Sep 18;6(3):918–34. doi: 10.3390/genes6030918 (PMC4584336; doi:10.3390/genes6030918)
Supplement: Supplementary File 1 [file genes-06-00918-s001.zip › genes-91449-supplementary-publish/genes-91449-supplementary figures.docx]

**Supplementary Materials**


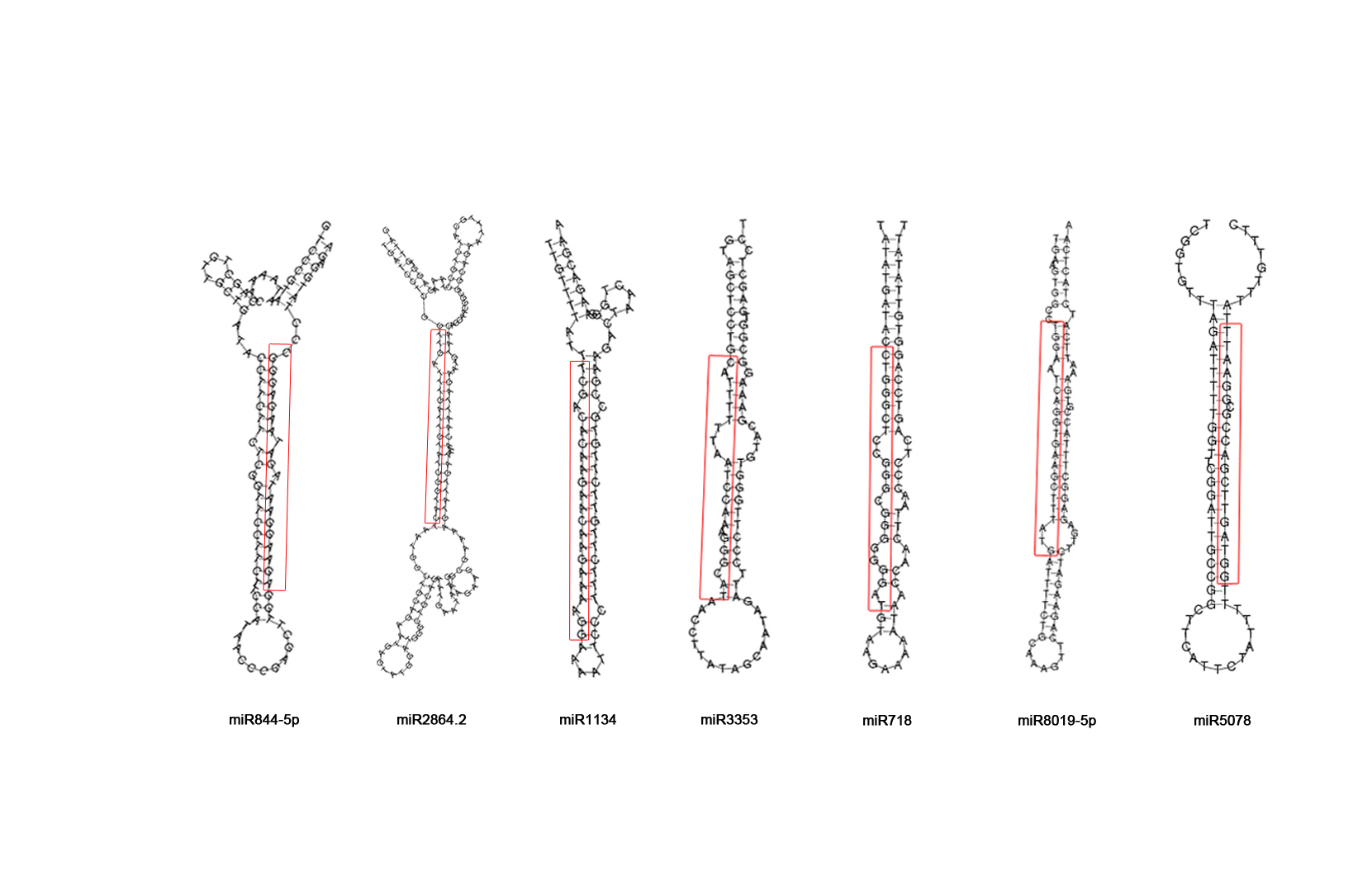


**Figure S1.** The secondary structures of some conserved pre-miRNAs in “Zifengyu”.


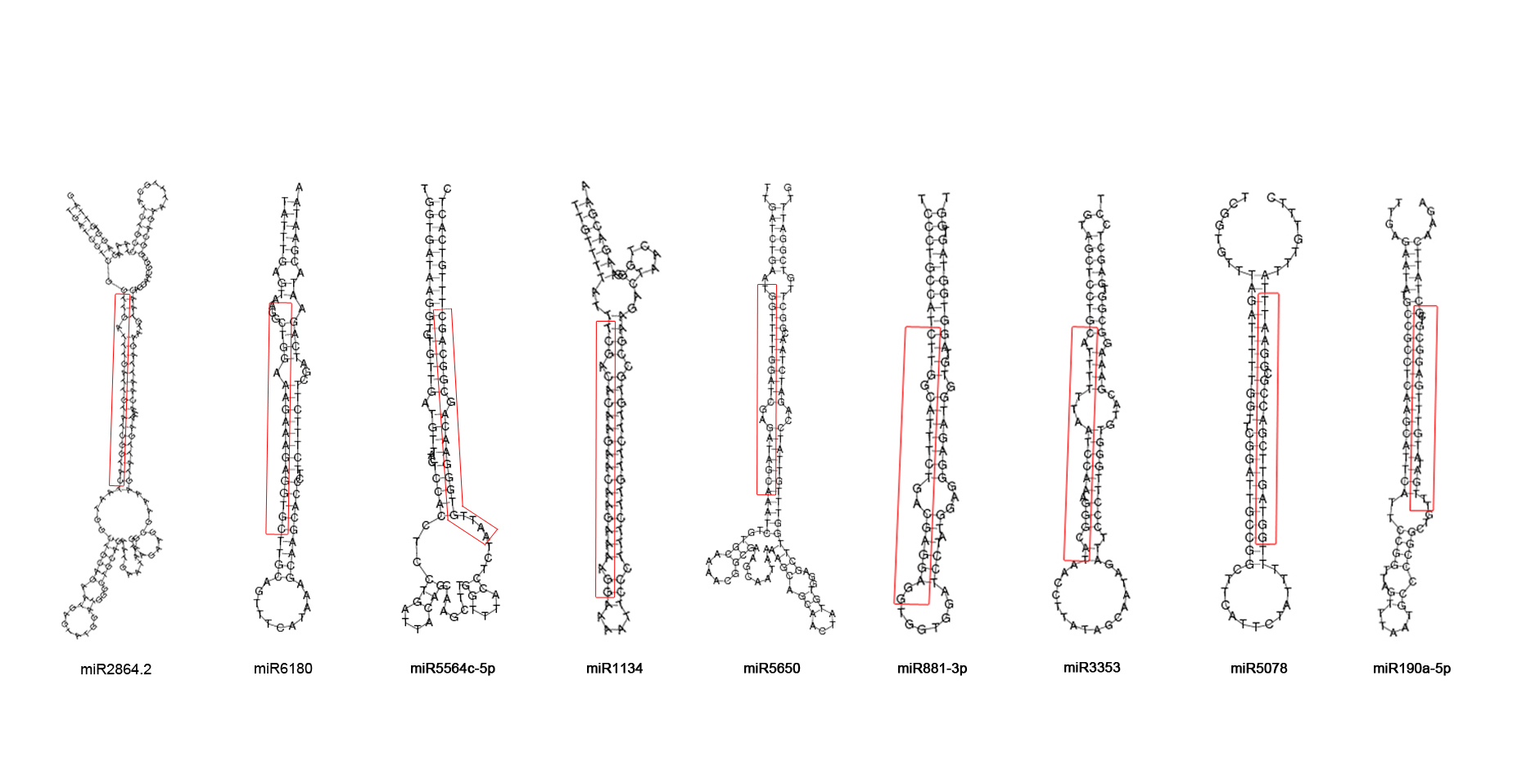


**Figure S2.** The secondary structures of some conserved pre-miRNAs in “Dafugui”.


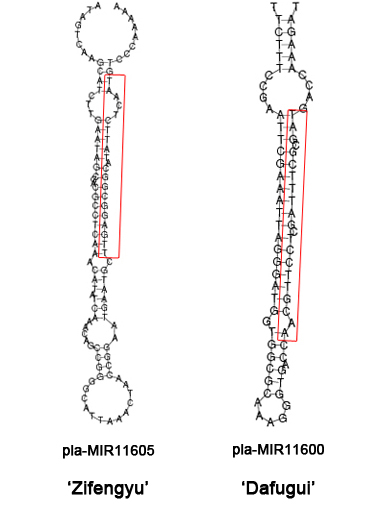


**Figure S3.** The secondary structures of some novel pre-miRNAs in “Zifengyu” and “Dafugui”.

© 2015 by the authors; licensee MDPI, Basel, Switzerland. This article is an open access article distributed under the terms and conditions of the Creative Commons Attribution license (http://creativecommons.org/licenses/by/4.0/).
